# Recurrent giant cell tumour of the thoracic spine managed by total *en bloc* spondylectomy and denosumab therapy: a case report

**DOI:** 10.1186/s12891-020-3129-4

**Published:** 2020-02-15

**Authors:** Ping-Guo Duan, Yong-Hong Sheng, Chang-Hao Deng, Ben-Yu Tang, Hao-Qun Yao

**Affiliations:** Department of Orthopaedic Surgery, The First Affiliated Hospital of Nanchang University, Nanchang, 330006 Jiangxi People’s Republic of China

**Keywords:** Giant cell tumour, Total *en bloc* spondylectomy, Thoracic spine, Denosumab, Recurrence

## Abstract

**Background:**

Giant cell tumour (GCT) of the bone is a rare, invasive benign bone tumour, which typically originates in the metaphyseal ends of long bones and rarely in the spine. Here, we report a rare case of recurrent GCT of the thoracic vertebra, which was managed by three-level total *en bloc* spondylectomy (TES) after denosumab therapy.

**Case presentation:**

A 50-year-old woman presented with a 2-month history of progressive lower back pain. Magnetic resonance imaging revealed destruction of the T11 vertebra and a soft tissue mass. The patient underwent tumour resection. Computed tomography at the 2-year follow-up revealed relapse of the resected tumour, which had spread to the T12 vertebral body. Subsequently, denosumab therapy was administered to the patient for 1 year. The growth of the tumour was controlled, and its boundary line was clear. Thereafter, TES for the T10-T12 vertebrae was performed, and spinal reconstruction was completed through a one-stage single posterior approach. The patient’s condition improved postoperatively, and no evidence of recurrence of GCT of the bone or spinal deformity was observed at the 32-month follow-up.

**Conclusions:**

Denosumab therapy contributed to tumour regression. Three-level TES may be an effective and feasible strategy for managing large recurrent GCTs of the spine after denosumab therapy.

## Background

Giant cell tumour (GCT) of the bone is a rare, invasive benign bone tumour, accounting for approximately 5% of primary bone tumours. It typically originates in the metaphyseal ends of long bones and rarely in the spine [1–3]. Approximately 1.4–9.4% of GCTs occur in the vertebrae above the sacrum in patients aged 20–40 years, and they more commonly occur in women than in men [4]. Although GCT is predominantly considered as a benign lesion, it may change from an indolent and static tumour to a locally invasive lesion with extensive bone destruction, cortical breakthroughs, and soft tissue expansion [5, 6].

Surgical treatment is the foundational treatment strategy for spinal GCT of the bone with the aim of preserving functionality, relieving pain, controlling local recurrence, and promoting prolonged survival [4]. Although intralesional curettage has been established as the preferred treatment for most GCTs, recommendations on treating tumours with rare localisations, such as in the spine or the sacrum, are still unclear [7, 8]. By comparison, total *en bloc* spondylectomy (TES) generally reduces local tumour recurrence and is currently a widely accepted surgical procedure for spinal tumours [9, 10].

Spinal GCT has a high recurrence rate of approximately 25–50% after surgical treatment. Therefore, reducing recurrence is the key to treatment [9, 11]. Denosumab is a human monoclonal antibody that specifically inhibits the receptor activator of nuclear factor-κB ligand (RANKL) by mimicking osteoprotegerin (OPG) that binds to RANKL, which in turn prevents RANKL from binding with the receptor activator of nuclear factor-κB (RANK) receptor, thereby inhibiting osteoclast activation. Denosumab has provided good clinical results [11–13]. Herein, we report a rare case of a recurrent large GCT of the thoracic spine that was successfully removed using three-level TES after denosumab therapy.

## Case presentation

A 50-year-old woman was admitted to our hospital in June 2013 owing to back pain radiating to the lower left abdomen for 2 months with progressive exacerbation. Physical examination revealed a tender point on the back, paraparesis with motor strength of 4/5 in both lower limbs, and decreased left inferior abdominal wall reflex. Magnetic resonance imaging (MRI) revealed destruction of the T11 vertebra and a soft tissue mass. To prevent rapid neurological deterioration owing to tumour growth, local curettage was planned using the posterior approach. However, pathologic examination of the neoplastic specimen using instant frozen section showed that the tumour was more likely to be malignant; thus, the involved vertebral body and upper and lower intervertebral discs were completely resected. Thereafter, spinal reconstruction was performed with a screw system and titanium mesh.

**Fig. 1:**
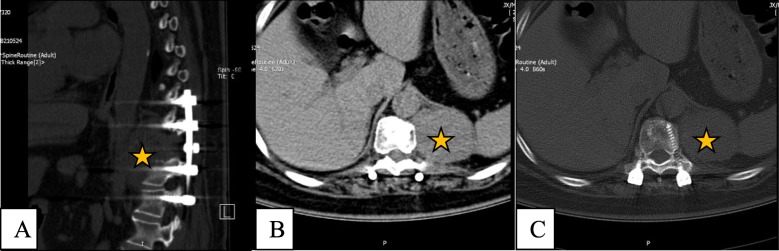
CT examination of the operative site (star indicated) 14 months after surgery. (**a**) CT sagittal view; (**b**) and (**c**) CT axial views

Follow-up computed tomography (CT) showed tumour recurrence at 14 months after surgery (Fig. 1a-c). The patient was advised to undergo surgical treatment again, which she refused owing to lack of obvious discomfort.

**Fig. 2:**
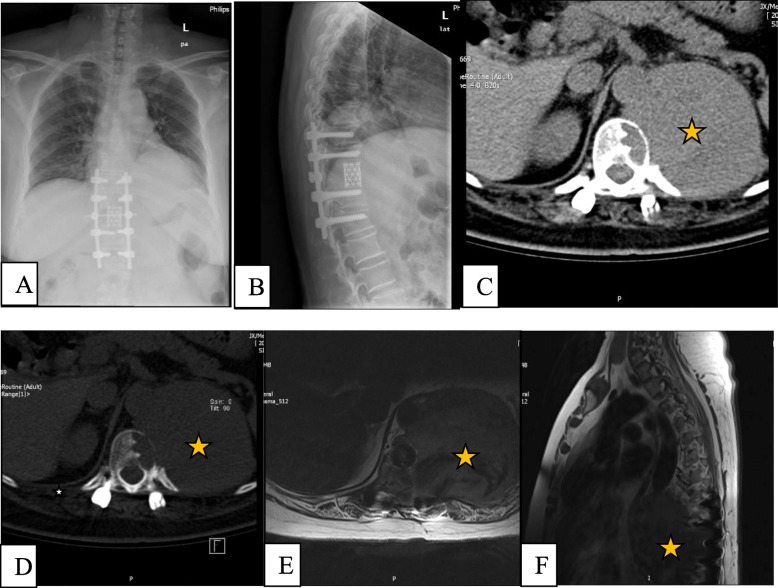
The images of radiograph, CT and MRI for thoracolumbar spine at 20 months after surgery. (**a**) Anteroposterior and (**b**) lateral radiographs; (**c**) and (**d**) CT axial views; (**e**) axial T2-weighted MRI; and (**f**) sagittal T1-weighted MRI. The images show local recurrence of a thoracic spinal giant cell tumour involving the T12 vertebral bodies, as indicated by the orange star

**Fig. 3:**
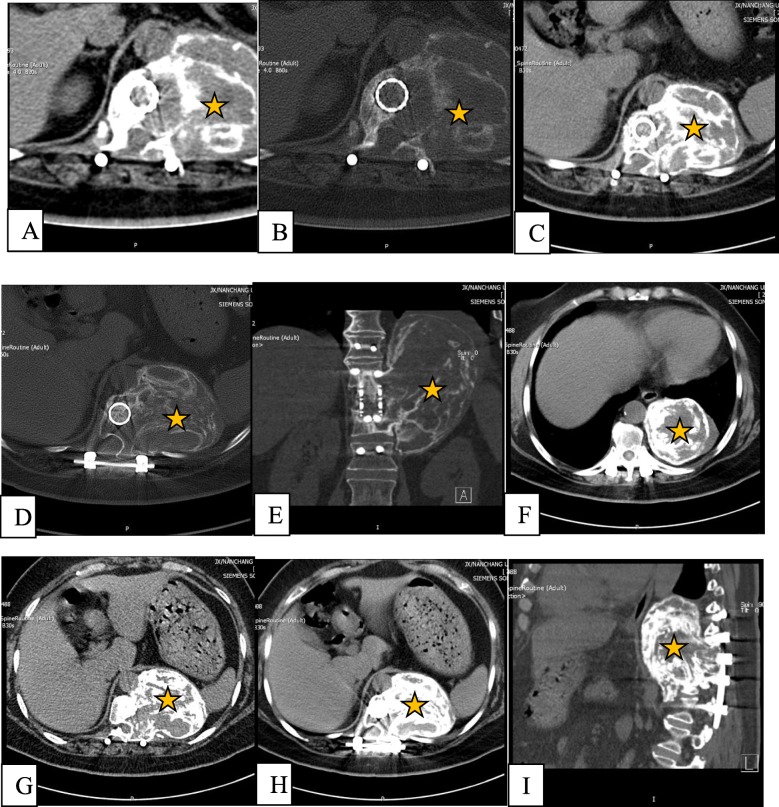
CT scan of the thoracolumbar spine after denosumab therapy. Images **a** and **b** were obtained in July 2015; images **c**, **d**, and **e** in November 2015; and images **f**, **g**, **h**, and **i** in June 2016. These images show the calcification of the tumour and shrinking after denosumab therapy

Twenty months after surgery, the patient was readmitted owing to back pain. On admission, radiography, CT, and MRI showed lytic bone destruction at the left edge of the T12 vertebra with a huge soft tissue mass shadow in the left thoracic cavity (Fig. 2a-f). The tumour volume was too large to be safely removed; thus, conservative treatment of denosumab was performed. After 1 year of denosumab therapy, the tumour growth was controlled, and its edges from the T10 to the T12 vertebral body were markedly calcified, and its boundary line became clear (Fig. 3a-i).

**Fig. 4:**
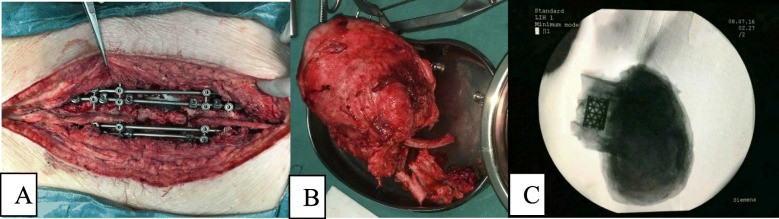
The tumour was removed successfully by three-level TES. (**a**) Intraoperative image. (**b**) Specimen of the *en bloc*-resected spinal GCT involving T10, T11, and T12. (**c**) Radiographs of the excised tumour

**Fig. 5:**
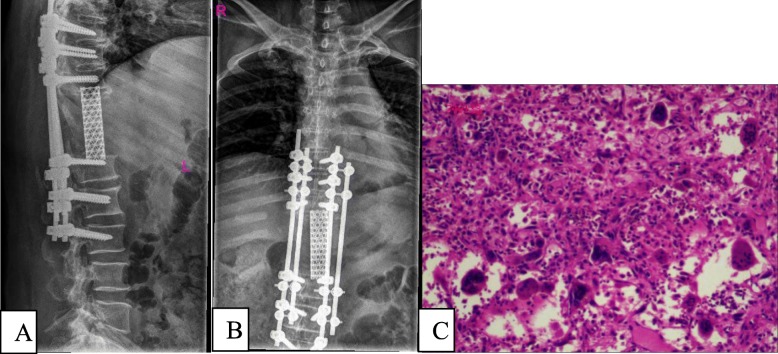
The images of radiograph and histopathology after three-level TES. (**a**) Lateral and (**b**) anteroposterior radiographs of the thoracolumbar spine showing a good implant position at 2 weeks after the surgery. (**c**) The pathological section slice shows patchy distribution of monocytes and giant cells among the mature and fused trabecular bone. The giant cells are distributed evenly, including some inflammatory cells, and some areas are necrotic

To prevent tumour recurrence, three-level TES was performed after 16 months of denosumab therapy. A 50-cm incision was made from T7 to L3 at the posterior median line. Twelve pedicle screws of appropriate length were implanted on both sides of the T7–9 and L1–3 vertebrae. The ribs of the right T10-T12 and the left T7-T12 were excised, and tumour tissues were separated. The T9-T10 and T12-L1 intervertebral discs were excised to divide the tumour, and the tumour and spinal tissues at the T10-T12 level were extracted from the left side of the spine (Fig. 4a-c). The tumour was approximately 20 × 15 cm in size, pale yellow, and hard. After resection of the tumour tissue, the left lung and diaphragmatic muscle tissues were explored and found to be severely compressed by the tumour. Some lung and diaphragmatic tissues were damaged. During the surgery, the thoracic surgeons performed emergency repair of the lung and diaphragmatic tissues. Next, two titanium rods were placed on both sides of the T7-L3 vertebrae. The cylindrical titanium mesh with appropriate length implanted into the autologous ilium was placed between T9 and L1. The pathological diagnosis was GCT of the bone (Fig. 5a-c).

Six-month postoperative radiographic examination revealed that the implant was in a good position and showed no loosening. The patient was instructed to wear a thoracolumbar brace for 3 months postoperatively.

**Fig. 6:**
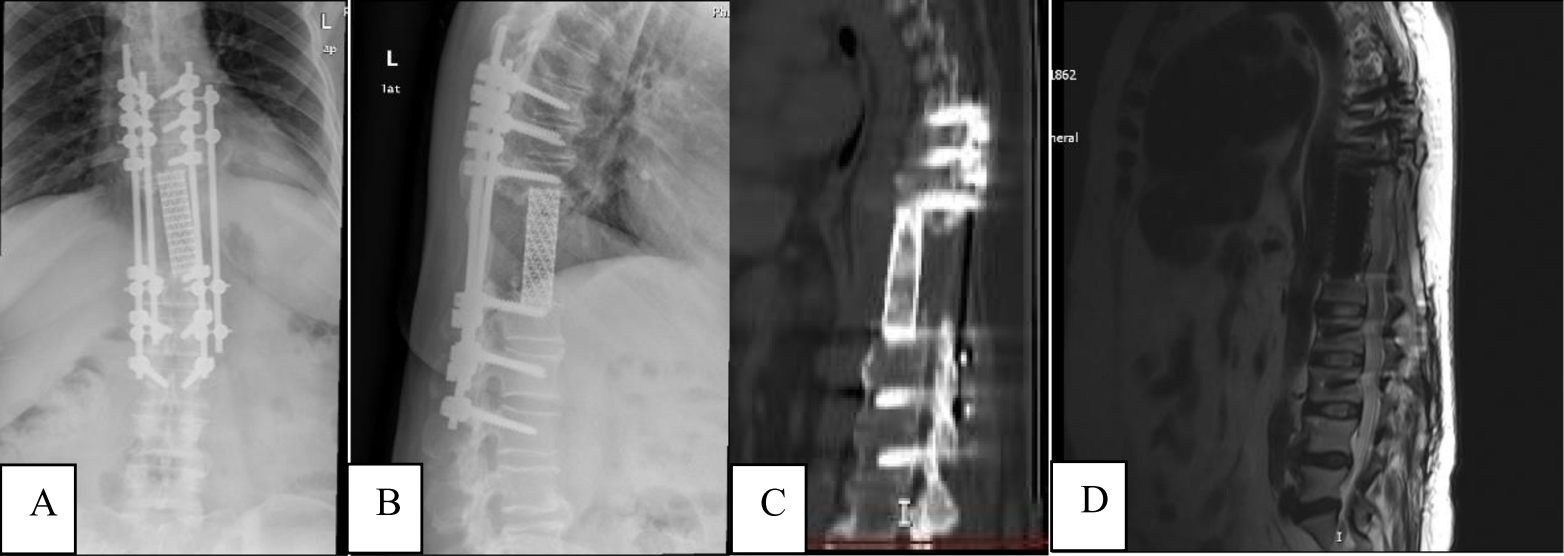
The images of radiograph, CT and MRI for thoracolumbar spine at 32 months after TES. (**a**) Anteroposterior and (**b**) lateral radiographs; (**c**) three-dimensional CT scan; and (**d**) MRI of the thoracolumbar spine showing a stable construct, biological fusion, and the absence of local recurrence

At the last follow-up (32 months after the second surgery), the patient had recovered well and could work normally. Moreover, no local tumour recurrence was observed on MRI and radiography, and three-dimensional CT showed successful biological reconstruction of the spine (Fig. 6a-d).

## Discussion and conclusions

GCT was first described by Cooper and Travers in 1818, and it mainly occurs in the femur, tibia, and radius, accounting for 55% of the lesions, but rarely originates from the vertebra above the sacrum [8, 14]. Spinal GCT is usually located in the vertebral body as opposed to the posterior elements; however, it is rarely confined to the vertebral body, and it continues to grow and may extend to involve the laminae, spinous process, and even the paravertebral area [2, 8, 14, 15]. As an invasive bone tumour, the postoperative recurrence rate of GCT is higher, and distant metastasis might occur. Approximately 1–4% of patients have lung metastasis [12, 16]; however, there was no pulmonary metastasis in the present case.

GCTs of the spine are reported to be expansile lytic lesions, with pain caused by a stretched periosteum being the most common manifestation, followed by pathological fracture (41%) and neurologic symptoms (32%) [15–17]. Diagnosis may be delayed, because back pain is a very common symptom and can be easily misdiagnosed [3].

However, most of these tumours are benign, and only a small number of GCTs (1–2%) may undergo malignant transformation, leading to a poor prognosis. According to the previous reports, GCTs can transform into fibrosarcoma, osteosarcoma, malignant fibrous histiocytoma, undifferentiated high-grade pleomorphic sarcoma, and undifferentiated sarcoma [4, 18, 19]. GCTs of the bone appear as expansive lytic lesions with non-sclerosing, well-defined edges on radiography, whereas CT and MRI provide information on the extent of the bone, bone marrow, and surrounding soft tissue involvement. MRI differentiates the lytic lesions from infectious spondylitis or postoperative complications, such as infections [20]. However, aspiration biopsy guided by CT is still needed to make a definite diagnosis of GCT of the bone [2, 15]. Histologically, GCT of the bone shows osteoclast-like giant cells [21]. Ewing’s sarcoma is another invasive bone lesion, with typical histopathological features of uniform round cells and irregularly shaped chromatic nuclei surrounded by a scanty cytoplasm [22].

Owing to the complexity of the spinal anatomy, the treatment of GCT of the spine has become a huge challenge. Intralesional curettage and *en bloc* resection are the most commonly used surgical methods; the former causes minor trauma with a high recurrence rate (27–65%), while the latter causes major trauma, often resulting in permanent nerve injury, with a low recurrence rate (0–12%) [3, 7, 12]. Although the Spine Oncology Study Group has conducted a systematic review of the literature in 2009 and strongly recommended that total *en bloc* resection of GCT of the spine was technically feasible, this recommendation was based on some very low-quality evidence and consensus among some experts [17]. The method of resolving local recurrence after surgery is still key to the treatment of GCT of the spine. This problem was addressed with the emergence of denosumab.

Histologically, GCT of the bone contains osteoclast-like giant cells that express RANK and stromal cells that express RANKL, a key mediator of osteoclast formation, activation, function, and survival. Excessive secretion of RANKL causes an imbalance in bone remodelling in favour of bone breakdown [21]. Denosumab is a fully human monoclonal antibody that inhibits RANKL; through its high affinity and specific binding to RANK, denosumab prevents the interaction between RANKL and RANK in a manner similar to that of OPG, thereby inhibiting bone absorption [21]. Branstetter et al. reported a phase II clinical study of 17 patients with GCT, showing that denosumab significantly reduced or eliminated RANK-activated GCTs, reduced the proportion of proliferative stromal cells in lesions, and increased the proportion of non-proliferative well-differentiated new bone tissue [8]. Other studies have also showed that denosumab could provide an objective tumour response rate of 72–86%, promoting tumour shrinkage and calcification [3, 18]. These reports suggest that denosumab might be helpful in the treatment of GCT of the bone. In the present case, the recurrent GCT appeared to have a similar response to denosumab, which could facilitate the performance of three-level TES.

In conclusion, the present report highlights a rare case of a large recurrent GCT in the thoracic spine, which was managed using three-level TES and denosumab therapy. Denosumab therapy contributes to tumour regression. TES may be an effective and feasible strategy for managing huge recurrent GCTs of the spine after denosumab therapy.

## Data Availability

The datasets used and/or analysed during the current study are available from the corresponding author on reasonable request.

## Abbreviations

CT: Computed tomography
GCT: Giant cell tumour
MRI: Magnetic resonance imaging
OPG: Osteoprotegerin
RANK: Receptor activator of nuclear factor-κB
RANKL: Receptor activator of nuclear factor-κB ligand
TES: Total *en bloc* spondylectomy
